# The validation of the Barcelona Orthorexia Scale—Spanish version: evidence from the general population

**DOI:** 10.1007/s40519-023-01616-6

**Published:** 2023-10-27

**Authors:** Anna Navarro, Carmen Varela, Adela Fusté, Ana Andrés, Carmina Saldaña

**Affiliations:** 1 Departament de Psicologia Clinica i Psicobiologia, Facultat de Psicologia, Universitat de Barcelona, Passeig Vall d’Hebron, 171, 08035 Barcelona, Spain; 2 Departmento de Ciencias de la Salud, Facultad de Ciencias de la Salud, Universidad de Burgos, Burgos, Spain; 3 Facultat de Psicologia, Ciències de l’Educació i de l’Esport, Blanquerna, Universitat Ramon Llull, Barcelona, Spain

**Keywords:** Orthorexia nervosa, BOS, Orthorexia nervosa assessment, Validation analysis, Factor analysis

## Abstract

**Purpose:**

To validate the Spanish version of Barcelona Orthorexia Scale (BOS) in general population, analyzing its items and both its internal structure and psychometric properties (internal consistency and temporal stability). In addition, the relationship between ON and external measures of attitudes towards food was assessed.

**Method:**

The general population sample consisted of 446 women and 104 men, aged between 18.31 and 69.44 years (*M* = 36.03; SD = 12.46). Of these, 39 participants answered again the questionnaires after one month from the first application. The assessment instruments were a sociodemographic questionnaire, the BOS, the Eating Attitudes Test-26 (EAT-26) and the Dutch Eating Behavior Questionnaire (DEBQ).

**Results:**

The final version of the BOS is composed of 35 items. Exploratory factor analysis extracted an internal structure of 5 factors (*Behavioral*, *Concern for healthy food*, *Attitudes and beliefs about food*, *Vital achievement* and *Emotional discomfort*). The BOS-35 and the factors presented good internal consistency (*α* = .80–.90), and an adequate temporal stability (*r* = .62–.88). The highest association was observed between the Emotional Distress (BOS) and the Diet subscale (EAT-26; *r* = .51).

**Conclusions:**

This first validation of the BOS has shown adequate psychometric properties, being a valid and reliable instrument to assess ON in the general population.

**Level of evidence** Level II: Evidence obtained from well-designed controlled trials without randomization.

**Supplementary Information:**

The online version contains supplementary material available at 10.1007/s40519-023-01616-6.

## Introduction

According to the consensus document on the definition and diagnostic criteria for orthorexia nervosa (ON) [1], this problem is characterized by its focus on food quality, overplanning of meals, and prioritization of nutritional value and perception of purity over taste [1–9]. This obsession with health is associated with inflexible beliefs and excessive control of their eating behaviors [4, 9, 10]. A transgression of self-imposed rules is followed by emotions like guilt, low self-esteem or anxiety [1, 5]. The main consequences of ON are the elimination of all foods considered unhealthy accompanied by physical and mental health complications [3, 5, 7, 11–13]. Previous studies have observed a resemblance between ON and obsessive–compulsive disorder (OCD), both presenting high perfectionism, excessive control, and anxiety [14–16]. A previous review provides a complete and integrative explanation of the multiple categories involved in the appearance of ON [17]. This review provides a comprehensive view of the etiology of this problem beyond the individual characteristics such as food choices, the perception and relation to the body, and the approach and regulation mechanism of the person. This approach includes the social level, which combines the education area, and the culture of professional and leisure activities, including exercise and sports. These categories have presented strong correlations with higher scores on the ON instruments. In addition, the social level includes the healthism concept, considered as the social construction of healthism. This concept is mainly observed in Western societies. People are encouraged through social media and advertising to be more aware of their health and the individual responsibility to achieve their health goals. However, there is another level that combines the other two categories (individual and social), called the societal level that introduces the concept orthorexic society, where ON occurs in societies where food perfectionism involves food organization, following by search and selection [17].

According to World Health Organization (WHO), a society focused on the promotion of healthy eating could be positive as a method to prevent diseases [2, 18]. However, an excessive attention to intake healthy foods, following a diet or excessive physical activity could lead to an obsession with healthy habits. An extreme focus on healthy eating is usually related with physical, psychological and social deterioration [3, 4, 19]. All of these factors are involved on the appearance, development and current definition of ON, although this problem has not been considered as an official diagnosis yet [3, 5, 10, 17, 19–21].

Therefore, ON has been identified as a problem with multiple causes and psychological consequences that could cause a significant distress and affect the social, work or family areas of the individual [1]. Moreover, the prevalence of ON has been increased, especially during the last decade. Two studies, carried out in Chine and Germany, respectively, used an instrument with diagnostic capacity for ON in college population. The results showed that the prevalence of ON was between 3.3 and 7.8%, being higher among male (10.6%) compared to female students (5.3%) [22, 23]. Another study with a sample of Spanish college population, assessed the presence of risk factors associated with the possible onset of ON, the estimated risk of developing this problem was between 9 and 17% [24]. There was another study carried out in Italy, also with university students, where the prevalence of ON was between 2.6 and 8.4% [20].

Knowledge about the etiology, prevalence, consequences and characteristics of ON has increased in recent years. However, due to the absence of an official diagnosis [20, 21], it seems necessary to design a valid and reliably instruments to assess ON [25, 26]. To date, several assessment instruments have been developed to assess ON in both general and clinical populations [27].

The first tool was the Bratman Orthorexia Test (BOT). This is a questionnaire of 10 dichotomous items to establish an early diagnosis of ON [28]. There are no studies in general population, but a validation has been carried out with German clinical population (the Ortho-10) [29].

The ORTO-15 was developed based on the BOT items to diagnose ON in general population [30]. Several validation studies of this tool have been conducted in different countries and populations. However, the psychometric properties obtained were not strong enough [31–37].

The Eating Habits Questionnaire-21 (EHQ-21) [38] was designed to assess ON symptoms obtaining good psychometric properties. However, there are no items to evaluate the emotions of people with ON after consuming food considered unhealthy or breaking self-imposed rules [35]. Another diagnose instrument is the Düsseldorfer Othorexie Skala (DOS) [39], composed by 10 items answered using a 4-Likert scale and with adequate psychometric properties. The Teruel Orthorexia Scale (TOS) [40] contains two factors (health orthorexia and orthorexia nervosa), that aim to differentiate between interest in following a healthy diet and pathological eating behavior.

Recently, two more tools were developed to assess ON, the Test of Orthorexia Nervosa-17 (TON-17) [41] and the Orthorexia Nervosa Inventory (ONI) [42]. Both instruments were developed to measure ON symptomatology, showing adequate psychometric properties and three different factors. TON-17 presented the subscales “Control of food quality”, “Fixation of health and healthy diet” and “Disorder symptoms” [41]. The factors founded in ONI were “Behaviors and preoccupation with healthy eating”, “Emotional Distress” and “Physical and Psychosocial Impairments” [42].

Access to valid and reliable instruments to assess ON is limited, mainly because these tools are based on initial descriptions of ON [26, 28]. It is necessary to contrast the description of diagnostic criteria and use the expert agreement methodology to define the items [9]. For that reason, prior to this investigation a new instrument was developed, the Barcelona Orthorexia Scale (BOS), both English and Spanish versions [43]. The difference with previous instruments is the evaluation of different clinical manifestations of ON without being a diagnosis tool. BOS includes the assessment of, among others, the characteristics presented on the individual level (i.e., food choices and the perception and relation to the body), as well as, the behaviors related to health goals [17].

The main aim of this study is the validation of BOS Spanish version and assess its psychometric properties in a Spanish sample of general population. The specific objectives were: (i) conduct an analysis of BOS items; (ii) assess the internal structure of the scale, and verify the suggested initial factors; (iii) analyze the reliability of the questionnaire in terms of internal consistency and the temporal stability of the BOS; and (iv) assess the relationship between BOS scores and eating attitudes using external measures. For the last objective, the relationship between BOS and the other instruments will be conducted to analyze the validity of the instrument, not to compare ON with another eating disorders.

## Method

### Development of BOS questionnaire

The BOS questionnaire was developed for the psychological assessment of the main variables of ON, using the Delphi methodology by experts’ agreement [43]. The initial number of suggested items was 105, divided into 6 different areas, as the authors' initial theoretical proposal: (1) cognitive area, (2) emotional area, (3) behavioral area, (4) negative consequences on health, (5) negative consequences on social or academic functioning, and (6) differential diagnosis with other eating disorders. The content of the items was analyzed through three rounds of Delphi study in which the participants (experts in the field of ON and in the field of eating disorders) valued the representativeness and clarity of each item as well as the correct location in the assigned areas. The sample consisted of 58 experts in the initial round and 30 in the final round from 17 countries. Items were added, modified or removed after the qualitative and quantitative analysis of the experts’ agreements and proposals for modifications. The final questionnaire consisted of 64 items with two identical versions in Spanish and English [43].

### Participants

A total of 550 participants from Spanish general population, obtained by random non-probability sampling, completed the study. Initially, questionnaires were administrated to 743 participants, but after verifying the following inclusion criteria: age between 18–70 years and the fulfilments of all questionnaires, the participation rate was 74%. Participants in the final sample were between 18.3 and 69.4 years old [mean (*M*) = 36.0; standard deviation (SD) = 12.5], and 81.1% of whom were women.

### Instruments

- Ad hoc sociodemographic questionnaire. Assessment of basic sociodemographic information like sex, age, ethnicity, marital status, education level, socioeconomic status and employment situation. Questions about weight and height were conducted to obtain the body mass index (BMI).
- Barcelona Orthorexia Scale (BOS). Instrument to assess the clinical manifestations of ON using 64 items answered by 5-Likert Scale (1 = Totally disagree; 5 = Totally agree) [43].
- Eating Attitudes Test-26 (EAT-26). Assessment of maladaptive attitudes and behaviors common on eating disorders, specifically, the symptoms of anorexia nervosa. A total of 26 items grouped in 3 subscales: Diet (13 items), Bulimia and Food Preoccupation (6 items) and Oral Control (7 items). Previous studies demonstrated an adequate internal consistency of this instrument (*α* = .80–.90) [43–47], including its Spanish validation [48].
- Dutch Eating Behavior Questionnaire (DEBQ). The instrument contains three scales to assess three possible eating behaviors: emotional intake, external intake and restrictive behavior. This tool presents high levels of internal consistency (*α* = .80–.90) [43, 49–52], including its Spanish validation [51].

The Spanish versions of the EAT-26 [48] and the DEBQ [51] were applied to analyze the convergent validity of the BOS, proving that the constructs that should be related show high correlations, for example Bulimia and Food preoccupation with Emotional Distress.

### Procedure

This study was approved by the Bioethics Committee of the University of Barcelona to apply to both clinical and general samples, although the research has been conducted only with general population. Participants were invited to participate through social media and snowball sampling was used to advertise the study. Also, some associations, nutrition and health federations, colleges of nutritionists, and psychological and nutrition centers collaborated with the diffusion of the study. After accepting informed consent, the participants accessed to the online questionnaires using an internet survey platform. The confidentiality of personal information was guaranteed, and no reward was given for participation. Participants were also required to participate in a second phase of the study. Those who provided their email addresses (*n* = 50) were contacted again 4 weeks later, in order to analyze the scale’s test–retest reliability. They were provided with the appropriate link via email to complete the second part of the study, Participation was also voluntarily and there was no reward. Originally, 50 participants were involved in the second part of the study, but only 39 participants completed the second administration (78% participation). Age range was 22.8–68.7 years (*M* = 35.0; SD = 14.1), and 74.4% were women.

### Data analysis

Analyses were performed using the program IBM SPSSS 24.0. A descriptive and quantitative analysis of the BOS items was conducted to select the final variables. To assess the properties of the items, analysis of inter-item correlation, corrected inter-item correlation (item homogeneity index), asymmetry and kurtosis were performed.

To analyze the internal structure of the BOS, an exploratory factorial analysis was conducted, and to estimate the factors, the method of unweighted least squares was chosen. To confirm the factor number, the selected criteria were the sedimentation graph, the parallel analysis (using the Monte Carlo PCA) [53], the theoretical bases and the interpretation of the solution. The direct *oblimin* rotation method was used to define the best factorial solution, selecting factors based on the number of variables loaded (minimum 3 variables per factor) and the factorial loading of each variable per factor (> .40).

An analysis of the internal consistency (Cronbach’s *α* and split half method) and the temporal stability (test–retest) were conducted in the final BOS version. Finally, the relationship between BOS and external measures (EAT-26 and DEBQ) were assessed using Pearson correlations, obtaining the convergent validity of the BOS.

## Results

Table 1 shows the sociodemographic variables of the sample.

**Table 1:** Sample sociodemographic variables

	Men (*N* = 104; 18.9%)	Women (*N* = 446; 81.1%)	Total (*N* = 550)
Age [*M*, (SD)]	38.2 (13.6)	35.6 (12.0)	36.0 (12.5)
Range of age	19.7–69.4	18.3–69.3	18.3–69.4
BMI (Kg/m^2^) [*M*, (SD)]	25.0 (3.0)	22.6 (3.5)	23.0 (3.5)
BMI [*N*, (%)]			
Underweight	1 (0.2%)	28 (5.1%)	29 (5.3%)
Normal-weight	56 (10.2%)	336 (61.1%)	392 (71.3%)
Overweight	42 (7.6%)	63 (11.5%)	105 (19.1%)
Obesity	5 (0.9%)	19 (3.5%)	24 (4.4%)
Education level [*N*, (%)]			
Elemental	4 (0.7%)	11 (2%)	15 (2.7%)
Secondary	21 (3.8%)	38 (6.9%)	59 (10.7%)
Superior	79 (14.4%)	397 (72.2%)	476 (86.5%)
Socioecomic status [*N*, (%)]			
No income	5 (0.9%)	59 (10.7%)	64 (11.6%)
< 1 MW	15 (2.7%)	78 (14.2%)	93 (16.9%)
1 MW–2 MW	20 (3.6%)	120 (21.8%)	140 (25.5%)
2 MW–3 MW	18 (3.3%)	99 (18%)	117 (21.3%)
3 MW–4 MW	18 (3.3%)	53 (9.6%)	71 (12.9%)
4 MW–5 MW	11 (2%)	16 (2.9%)	27 (4.9%)
> 5 MW	17 (3.1%)	21 (3.8%)	38 (6.9%)

*N*, sample size; *M*, mean; SD, standard deviation; MW, minimum wage

Reliability analysis of the initial BOS were conducted showing an excellent internal consistence (Cronbach’s *α* = .949) and good test–retest reliability (*r* = .876). After that, the 64 initial items were analyzed using inter-item and corrected inter-item correlations, showing 7 items with a low homogeneity index (< .35). Also, 19 items were removed due to excessive values (> 2) in asymmetry and kurtosis analysis. The final revised questionnaire consisted of 38 items.

### Internal structure of BOS

An exploratory factorial analysis (EFA) was conducted to analyze internal structure of the questionnaire with data from the general sample (*n* = 550). Kaiser–Meyer–Olkin coefficient (KMO = .950) and Sphericity Bartlett’s Test (*χ*^2^ = 13,153.6, d*f* = 730, *p* < .001) showed the appropriateness of the data for the EFA.

The unweighted least squares method was used to factor extraction, and according to sedimentation graph and parallel analysis the best factorial solution was the selection of the first five factors. The rotated solution was analyzed using the direct rotation (*Oblimin*) method. Table 2 shows the configuration matrix revealing a simple structure with 5 factors explaining the 59.6% of total variance (Factor 1 = 38.4%; Factor 2 = 8.6%; Factor 3 = 4.7%; Factor 4 = 4.1%; Factor 5 = 3.8%). It is observed the weight of each item for each factor and an aggrupation of items in one factor, without saturations in the other ones (> .40). Items 6, 9 and 22 were removed because their loads were low for all factors, the final version of the BOS was composed by 35 items (to observe original items from BOS-64 and remained items from BOS-35, see Supplementary information-Table S1).

**Table 2:** Factor loadings from exploratory factor analysis

Items	Factors				
	Factor 1 (B)	Factor 2 (PHF)	Factor 3 (ABF)	Factor 4 (VA)	Factor 5 (ED)
BOS2	− .005	**.670**	.210	.047	− .072
BOS3	.026	.080	**.406**	.204	− .051
BOS4	− .018	.077	**.423**	− .080	.272
BOS5	− .044	**.702**	.082	.011	.128
BOS6	.056	.398	.227	− .002	.118
BOS7	.217	**.417**	.275	− .133	.060
BOS9	.047	.381	.333	.180	− .153
BOS10	− .123	**.404**	.252	.030	.105
BOS11	.193	− .016	**.542**	− .033	.131
BOS12	.257	.020	**.579**	.079	.031
BOS13	.229	.133	**.522**	.041	.070
BOS14	.087	.093	**.430**	.173	.018
BOS15	.010	− .053	.171	− .035	**.700**
BOS16	.008	.079	.059	.042	**.717**
BOS17	.051	− .015	.113	.053	**.674**
BOS19	.013	.002	.296	− .041	**.500**
BOS20	.037	− .059	.056	.042	**.764**
BOS21	.007	.099	− .110	.030	**.762**
BOS22	.180	.040	.127	.009	.380
BOS23	.015	.009	− .141	.117	**.836**
BOS24	.046	.029	− .152	.126	**.809**
BOS25	.197	.171	− .064	.026	**.591**
BOS26	.336	− .006	.047	− .009	**.498**
BOS27	.100	.068	− .006	**.660**	.156
BOS28	.127	− .073	.134	**.678**	.099
BOS29	− .064	.116	.000	**.631**	.247
BOS30	− .066	.097	− .039	.307	**.605**
BOS31	.089	**.771**	− .094	.029	.031
BOS32	.058	**.897**	− .147	.000	.003
BOS33	.075	**.922**	− .140	− .009	.002
BOS34	**.570**	.018	.072	− .048	.100
BOS35	**.483**	.115	.089	− .098	.085
BOS39	.129	**.522**	.030	.090	.004
BOS40	**.548**	.149	− .061	− .046	.245
BOS41	**.418**	.342	.017	.071	.074
BOS42	**.658**	.074	.075	.014	.024
BOS43	**.598**	− .055	− .008	.182	− .026
BOS44	**.496**	.141	.054	.170	− .028

*ABF* Attitudes and Beliefs about Food*, B* Behavioral, *ED* Emotional Distress, *PHF* Preoccupation about Healthy Food, *VA* Vital Achievement

Method of extraction: unweighted least squares method. Rotation method: *Oblimin* with Kaiser normalization. Bold values indicate the superior factorial loading (> .40) in absolute values

The final factors were Factor 1 Behavioral (B), Factor 2 Preoccupation about Healthy Food (PHF), Factor 3 Attitudes and Beliefs about Food (ABF), Factor 4 Vital Achievement (VA) and Factor 5 Emotional Distress (EM). Factors 1, 3 and 5 agree with the behavioral, cognitive and emotional areas originally proposed. Factor 2 included items from the original behavioral and cognitive areas. Finally, Factor 4 presented items included in the original emotional area but relevant for the orthorexia construct.

The correlations between factors were assessed. Table 3 shows the correlation matrix between the 5 factors, presenting Factors 1 and 2 higher correlations with the other factors.

**Table 3:** Factorial correlations

Factors	*B*	PHF	ABF	VA	ED
*B*	–				
PHF	.514	–			
ABF	.389	.428	–		
VA	.303	.344	.210	–	
ED	.496	.393	.323	.375	–

*ABF* Attitudes and Beliefs about Food*, B* Behavioral, *ED* Emotional Distress, *PHF* Preoccupation about Healthy Food, *VA* Vital Achievement

### Reliability of BOS

Table 4 shows an excellent internal consistency for both factors and the final version of the BOS, revealing coefficients of Cronbach’s *α* between .80–.94 and Spearman–Brown coefficients higher than .81. Also, all factors and the questionnaire present an adequate medium inter-items correlation (*r* > .35).

**Table 4:** Descriptive data and coefficients of internal consistency for each factor and BOS final version

Factors	Items number	*N*	*M*	ST	*α*	95%CI	Mean inter-items *r*
B	7	550	13.4	5.7	.851	.83–.87	.449
PHF	8	550	22.0	7.5	.902	.89–.91	.532
ABF	6	550	17.8	4.6	.804	.78–.83	.408
VA	3	550	9.4	3.3	.836	.81–.86	.629
ED	11	550	22.8	9.6	.935	.93–.94	.568
Total	35	550	85.4	24.8	.953	.95–.96	.367

*ABF* Attitudes and Beliefs about Food*, B* Behavioral, *CI* Confidence Interval, *ED* Emotional Distress, *M* Media *N* Sample Size, *PHF* Preoccupation about Healthy Food, *r* correlation*, ST* Standard Deviation, *VA* Vital Achievement

### Factors and BOS final version stability

Table 5 shows the final version of the BOS, a high reliability test–retest (*r* = .877) was reported, finding a significant correlation between the two administrations (*p* < .001). The five factors also show high reliability coefficients (.624–.818).

**Table 5:** Descriptive data and temporal stability coefficients for each factor and BOS final version

Factors	Items number	*N*	Time 1		Time 2		*r* Test–retest
			*M*	ST	Md	ST	
B	7	39	10.7	4.4	11.0	3.9	.752
PHF	8	39	17.1	5.1	16.8	4.6	.818
ABF	6	39	15.8	4.3	15.2	4.0	.809
VA	3	39	8.6	2.8	8.6	3.1	.624
ED	11	39	20.4	9.3	19.6	7.6	.800
BOS	34	39	72.67	22.70	71.1	19.4	.877

*ABF* Attitudes and Beliefs about Food*, B* Behavioral, *BOS* Barcelona Orthorexia Scale, *ED* Emotional Distress, *M* Media *PHF* Preoccupation about Healthy Food, *r* correlation, *ST* Standard Deviation, *VA* Vital Achievement. The time between time 1 and time 2 was 1 month

### Relationship between BOS scores and eating disorder symptomatology

Finally, Table 6 presents the correlations between the five BOS factors and the subscales of EAT-26 AND DEBQ. In general, it has been observed very low, low, and moderated correlations from *r* = .101, *p* < .05 to *r* = .51, *p* < − 01. The five factors of the BOS correlate more significantly with the EAT-26 subscales than DEBQ subscales. The most significant associations were observed between Emotional Distress (BOS) and Diet (EAT-26) (*r* = .510, *p* < .01), and between Emotional Distress (BOS) and Bulimia and Food Preoccupation (EAT-26) (*r* = .508, *p* < .01).

**Table 6:** Correlations between five BOS factors and EAT-26 and DEBQ subscales

	*B*	PHF	ABF	VA	ED
EAT-26 (diet)	.449**	.314**	.259**	.348**	.510**
EAT-26 (bulimia and food preoccupation)	.413**	.459**	.305**	.334**	.508**
EAT-26 (oral control)	.382**	.302**	.254**	.246**	.332**
DEBQ (emotional intake)	.153**	.101*	.050	.250**	.362**
DEBQ (external intake)	− .056	− .062	− .083	.205**	.168**
DEBQ (restrictive intake)	.364**	.300**	.262**	.351**	.489**

*ABF* Attitudes and Beliefs about Food*, B* Behavioral, *DEBQ* Dutch Eating Behavior Questionnaire; *EAT-26* Eating Attitudes Test-26, *ED* Emotional Distress, *PHF* Preoccupation about Healthy Food, *VA* Vital Achievement

^*^p < .05 **p < .01

## Discussion

The main objective of this study was to validate the Spanish version of the BOS in the general population. The BOS was intended to assess the psychological variables of ON without being a diagnostic instrument.

The first specific objective of this study was to analyze the original 64 items suggested by the experts [43]. Item analysis revealed that 26 items had to be removed. These items were primarily classified into the original areas of differential diagnosis and negative consequences on health, social or academic functioning. Although the assessed aspects are theoretically relevant, their presence is associated with those cases where ON is causing significant discomfort in various areas of the person's life over a long period of time. However, behavioral, cognitive, and emotional symptoms represent core aspects of ON [5, 6, 11, 13]. Therefore, these aspects are relevant for early identification and linked to the main objective of the BOS, the evaluation of psychological variables of ON. The social and societal aspects involved on the onset of ON [17], should be considered in instruments designed for diagnosis. The differential diagnosis items were a distinctive element of the BOS compared to other questionnaires but in the clinical practice there are other instruments to assess eating disorders. For that reason, the use of instruments like EAT-26 and DEBQ is relevant to analyze the validity of the BOS [43].

In the present study the remaining 38-item version of the original BOS was used to analyze its internal structure. According to the second specific objective of the study, the five-factor solution was the most appropriate to explain the variance of the questionnaire. These five factors are correlated representing a multidimensional structure equal to the original version. After item analysis, three more items were removed due to low factor loading on all factors. A final version of 35-item v was obtained. The final factorial structure reflected the central symptomatology of ON (behavioral-cognitive-emotional). The factor “Preoccupation about healthy food” presented more correlations with the other factors, showing the obsession with healthy food as central characteristic of ON. The highest correlation between factors involved this factor and the “Behavioral” factor, supporting the association between the obsession and the behavioral rituals like the restriction of food considered unhealthy. This obsession with consuming only healthy foods could be related to the concept of “healthism”, overpromoted in social media and advertising in recent years. In relation to this concept, the “BOS-35” includes a scale called “Vital Achievement” [17]. The “Behavioral” factor also presented a high correlation with the “Emotional Distress” factor (*r* = .50). This correlation could explain the relationship between the ON behavioral pattern and maladaptive emotions of breaking self-imposed rules [1, 5–7].

The reliability of the final version of the BOS has shown excellent internal consistence and temporal stability. The individual factors also presented high reliability. All factors showed an adequate temporal stability except “Vital Achievement”. The possible explanation could be the reduced number of items to assess this factor. However, this factor was retained because of its theoretical relevance, the high saturations of the three items, and their high communalities.

The results of the associations between the BOS factors and the external measures (subscales of the EAT-26 and the DEBQ) were similar to the results found in other validations studies [35, 38]. The most significant correlations were between “Emotional Distress” (BOS) and the subscales “Diet” and “Bulimia and Food preoccupation” (EAT-26). Other significant correlations were found between “Preoccupation about healthy food” (BOS) and “Bulimia and Food Preoccupation” (EAT-26); and “Behavioral” (BOS) and “Diet” (EAT-26). These associations were expectable because assess similar aspects related to pathological eating behaviors [37, 38]. However, the correlations between BOS and DEBQ were low and/or non-significant, except for moderate-low correlations between “Emotional Distress” (BOS) with “Restrictive Intake” and “Emotional Intake” (DEBQ); and the “Behavioral” factor (BOS) and “Restrictive Intake” (DEBQ). The lower correlations values between BOS and DEBQ compared with EAT-26, could be explained because BOS assesses aspects of ON symptomatology and DEBQ specific eating behaviors [43, 49–52]. According to previous studies, the results of this study have shown a moderated association between ON assessed by BOS and symptomatology eating disorders measured using EAT-26 and DEBQ [11, 13, 54]. Therefore, the results confirmed BOS assesses the characteristics of pathological eating, providing clinical evidence of ON as disordered eating. However, further investigation is needed, because there is no agreement about the conceptualization of ON attending to previous researchers [11, 15].

This study has some limitations. First, the distribution by sex in the sample was unbalanced. In the future, to improve the representativeness of the sample, more male participants should be recruited, whereas some of the studies providing prevalence data [22, 23] indicate that ON is more prevalent among men than among women. For that reason, the sampling method is considered another limitation and should be more controlled in future studies. Furthermore, the sample size for the test–retest analysis was small and the results should be interpreted with care. Another limitation was the absence of another ON questionnaire with good psychometric properties in Spanish, where the sample of the present study was recruited. This was an obstacle to the BOS convergent validity analysis. Moreover, the difference between healthy eating and the obsession about food is not well-established in current society [1], promoting certain social desirability in the participants. Participants could respond thinking that characteristics or manifestations of ON are non-prejudicial to their health, due to the limited information about this problem.

However, this investigation also presents some strengths. This study is the first validation of a Spanish instrument for ON in general population. The size of the recruited initial sample was enough to consider stable de factorial solution and the results about the psychometric properties of the BOS. One of the most relevant strengths of the study is to have achieved a reduced version of the BOS, 35 items compared to the original 64 items, with 5 factors with good psychometric properties. The content of the items of the different factors will provide very useful information both for developing prevention programs and for creating intervention programs. The use of the BOS-35, without been a screening or diagnostic questionnaire, will provide clinicians with accurate information about the ON characteristics of their clients. For example, they will be able to address maladaptive behaviors related to food selection, cooking and related eating behavior in therapy; they will also be able to have information about cognitive distortions specific to people with ON and restructure them to reduce the emotional discomfort they generate. Finally, the brevity of the questionnaires is highly valued and of interest to researchers and clinicians, as it allows participants to respond with less time and emotional cost. To sum up, this study provided the first validation of the Spanish version of the BOS, showing good psychometric properties, providing a new tool for this emerging problem.

### What is already known of this subject?

ON is emerging as a new eating problem. Its clinical evidence has promoted the appearance of different instruments to measure this construct in different countries and languages. The design of the BOS has been explained in a previous study, obtaining promising and positive results.

### What does this study add?

The first Spanish validation of a new tool to assess ON in general population. Providing a final version of the BOS with adequate psychometric properties. The results obtained in this study support the use of the Spanish version of BOS as a valid and reliable of ON symptomatology in general population.

### Supplementary Information

Below is the link to the electronic supplementary material.

Supplementary file1 (DOCX 17.5 KB)

## Data Availability

The datasets generated during and/or analyzed during the current study are available from the corresponding author on reasonable request.
