# Supplementary material for: The validation of the Barcelona Orthorexia Scale—Spanish version: evidence from the general population
Source: Eat Weight Disord. 2023 Oct 27;28(1):90. doi: 10.1007/s40519-023-01616-6 (PMC10611628; doi:10.1007/s40519-023-01616-6)
Supplement: Supplementary file 1 — Supplementary file1 (DOCX 17.5 KB) [file 40519_2023_1616_MOESM1_ESM.docx]

**Supplementary Information**

**Table 1. Items from the original version, BOS-64, and items remained at validated version, BOS-35.**

|  | BOS -64 | BOS - 35 |
| --- | --- | --- |
| BOS1 | Cuidar la salud eligiendo bien los alimentos que se consumen debería ser una de las cosas más importantes para todo el mundo. | - |
| BOS2 | Me preocupo mucho más que la mayoría de personas por saber si los alimentos que ingiero son los más saludables para mí. | Remained |
| BOS3 | Comer sano debería ser el valor fundamental de esta sociedad. | Remained |
| BOS4 | Si como alimentos poco saludables, me preocupa que pueda ponerme enfermo/a. | Remained |
| BOS5 | Paso más tiempo que la mayoría de personas preocupándome por mi salud y por cómo mejorarla a través de la dieta. | Remained |
| BOS6 | Intento no infringir ninguna de mis normas dietéticas. | - |
| BOS7 | Necesito conocer todos los ingredientes de las comidas que como. | Remained |
| BOS8 | Creo que la mayoría de personas comen muchos alimentos poco saludables. | - |
| BOS9 | He aprendido a comer correctamente, y creo que es muy importante seguir las normas de una alimentación saludable en todo momento. | - |
| BOS10 | Me preocupa la influencia que tiene la dieta sobre mi salud. | Remained |
| BOS11 | Solamente debo comer alimentos saludables. | Remained |
| BOS12 | Comer sano es el valor más importante en mi vida. | Remained |
| BOS 13 | Alcanzar la perfección a la hora de elegir alimentos saludables es sumamente importante para mí. | Remained |
| BOS14 | Es muy importante mantener el organismo libre de alimentos poco saludables. | Remained |
| BOS15 | Siento vergüenza cuando como alimentos que no considero que sean saludables. | Remained |
| BOS16 | Me siento nervioso/a o ansioso/a cuando como alimentos que considero que no son saludables. | Remained |
| BOS17 | Me siento impuro/a cuando como alimentos que considero que no son saludables. | Remained |
| BOS18 | Siento asco cuando como cerca de personas que comen alimentos que considero muy poco saludables. | - |
| BOS19 | Me da miedo enfermar si como alimentos poco saludables. | Remained |
| BOS20 | Me siento fracasado/a, si como alimentos o comidas que no son saludables. | Remained |
| BOS21 | Pensar en incumplir alguna de mis normas dietéticas, hace que me sienta inquieto y/o culpable. | Remained |
| BOS22 | Siento repulsión hacia las personas que no se preocupan lo suficiente por seleccionar alimentos saludables. | - |
| BOS23 | Me siento culpable cada vez que como alimentos poco saludables. | Remained |
| BOS24 | Me siento mal conmigo mismo cuando como alimentos que no forman parte de mis normas dietéticas. | Remained |
| BOS25 | Me siento ansioso/a cuando no puedo preparar, consumir o seleccionar alimentos que se adapten a mis estándares dietéticos. | Remained |
| BOS26 | Siento asco cuando me veo expuesto/a a alimentos o comidas que considero que son poco saludables. | Remained |
| BOS27 | Me siento satisfecho/a y puro/a cuando controlo lo que como de acuerdo con mis creencias sobre la comida saludable. | Remained |
| BOS28 | Me siento perfecto/a cuando como alimentos que considero que son saludables. | Remained |
| BOS29 | Mi autoestima mejora cuando como alimentos saludables. | Remained |
| BOS30 | Cuando como alimentos poco saludables, mi autoestima empeora. | Remained |
| BOS31 | Dedico mucho más tiempo que la mayoría de personas a planificar lo que voy a comer. | Remained |
| BOS32 | Paso mucho más tiempo que la mayoría de personas leyendo sobre comida saludable. | Remained |
| BOS33 | Paso mucho más tiempo que la mayoría de personas seleccionando y adquiriendo alimentos saludables. | Remained |
| BOS34 | He eliminado uno o varios grupos de alimentos completos de mi dieta, como las legumbres, almidones, carnes, lácteos, grasas y aceites, alimentos cocidos, verduras solanáceas comestibles etc., porque no los considero saludables. | Remained |
| BOS35 | Solamente como alimentos que yo mismo/a he preparado en casa, para garantizar que sean saludables. | Remained |
| BOS36 | Solo soy capaz de comer alimentos en restaurantes u organizaciones que siguen mis teorías e ideas sobre una alimentación saludable. | - |
| BOS37 | Si cometo un desliz y como alimentos que no son saludables, tengo que tomar ciertas medidas para purificarme. | - |
| BOS38 | No me permito comer alimentos que considero no saludables. | - |
| BOS39 | Intento convencer a otras personas para que coman de forma saludable como yo. | Remained |
| BOS40 | Debido a mi preocupación por la comida sana, la lista de alimentos que puedo comer se ha ido restringiendo cada vez más. | Remained |
| BOS41 | Elijo los alimentos exclusivamente en función de lo sanos que son. | Remained |
| BOS42 | Desde que he cambiado mi forma de comer, he eliminado totalmente determinados grupos de alimentos porque considero que no son saludables. | Remained |
| BOS43 | Suelo comer los mismos alimentos y comidas cada día porque mi dieta actual es saludable. | Remained |
| BOS44 | Siempre sigo el mismo procedimiento para preparar mis comidas a fin de maximizar su valor nutricional. | Remained |
| BOS45 | Desde que empecé a comer alimentos más saludables, he tenido algunos problemas físicos, como anemia, o he presentado signos de malnutrición. | - |
| BOS46 | Puede ser que la dieta restringida que sigo me haya causado algunos problemas de salud. | - |
| BOS47 | Aunque la intención era que los cambios en mi dieta me hicieran estar más sano/a, puede ser que me hayan causado o empeorado algunos problemas de salud sin darme cuenta. | - |
| BOS48 | Algún profesional sanitario me ha dicho que mi dieta es demasiado restrictiva y que me está perjudicando. | - |
| BOS49 | Tengo algunos problemas, como pérdida de cabello, anemia, cambios en la piel, fatiga y mareos, que, en parte, pueden estar causados por la dieta. | - |
| BOS50 | La gente me dice que tengo mal aspecto y que he desarrollado signos de malnutrición. | - |
| BOS51 | Salgo menos a comer con mis amigos que antes, porque sus hábitos alimentarios no son saludables. | - |
| BOS52 | Mis decisiones respecto la alimentación, han afectado mi rendimiento laboral/académico. | - |
| BOS53 | Mis relaciones con amigos y/o familiares se han visto perjudicadas por mi preocupación por los hábitos alimentarios. | - |
| BOS54 | Ya no puedo compartir comidas con mis amigos y familiares, porque no habrá alimentos que pueda comer. | - |
| BOS55 | He sido objeto de críticas entre mi círculo de amistades a causa de mis hábitos alimentarios. | - |
| BOS56 | Me siento rechazado/a o infravalorado/a por mi entorno social debido a mis hábitos alimentarios. | - |
| BOS57 | Voy a menos reuniones con familiares y/o amigos porque no comparten mis ideas sobre la alimentación saludable. | - |
| BOS58 | Creo que mi dieta ha causado problemas en mi trabajo, escuela y/o en mis relaciones sociales. | - |
| BOS59 | Tengo que llevarme mi propia comida cuando voy a comer con otras personas, porque no habrá alimentos que pueda comer. | - |
| BOS60 | Presto atención a mi dieta para mantenerme delgado/a. | - |
| BOS61 | Tengo miedo de ganar peso. | - |
| BOS62 | Mi objetivo principal en relación con mis hábitos alimentarios es perder peso. | - |
| BOS63 | Me preocupa más mi salud que estar delgado/a. | - |
| BOS64 | Lo importante es tener un cuerpo saludable, no estar delgado/a. | - |

Note. BOS = Barcelona Orthorexia Scale. Shaded are all the items that remain in the BOS validation (35 items).
